# Hyper‐CVAD in Adults With Acute Lymphoblastic Leukemia in Ecuador: A Multicenter Retrospective Study

**DOI:** 10.1155/ah/9995923

**Published:** 2025-12-15

**Authors:** Brenner Sabando, Jairo Quinonez, Andrés Orquera, Danilo Navarrete, Jhoanna Ramírez, Lorena Sanchez, Teodoro Chisesi, Jorge Oliveros, María A. Pacheco, María C. Trujillo, Carlos Plaza, Yaira Loor

**Affiliations:** ^1^ Department of Hematology, IRHED Medical Specialties Center, Guayaquil, Ecuador; ^2^ Hematology Service, Carlos Andrade Marín Hospital, Quito, Ecuador; ^3^ Hemato-Oncology and Medicine Transfusion Department, Solca Cancer Institute, Manabi, Ecuador; ^4^ Hematology Service, Teodoro Maldonado Carbo Hospital, Guayaquil, Ecuador; ^5^ Department of Hematology, Abel Gilbert Ponton Hospital, Guayaquil, Ecuador; ^6^ Oncology Department, Angela Serra Association for Cancer Research, Lecce, Italy; ^7^ Hematology Service, Luis Vernaza Hospital, Guayaquil, Ecuador; ^8^ Hemato-Oncology and Medicine Transfusion Department, Solca Cancer Institute, Cuenca, Ecuador; ^9^ Department of Hematology, Eugenio Espejo Hospital, Quito, Ecuador, hee.gob.ec; ^10^ Hemato-Oncology and Medicine Transfusion Department, Solca Cancer Institute, Guayaquil, Ecuador

## Abstract

**Background:**

The Hyper‐CVAD protocol is a chemotherapy regimen widely used in hematological malignancies. It is considered one of most promising remission‐leading treatment option for adults with acute lymphoblastic leukemia (ALL).

**Objective:**

To determine the outcomes of adults with ALL treated with the Hyper‐CVAD protocol in Ecuador.

**Methods:**

This multicenter, retrospective, cross‐sectional study compared the outcomes of ALL patients aged 15 years and older treated with hyper‐CVAD protocol in 8 specialized centers.

**Results:**

139 patients with ALL treated with the Hyper‐CVAD protocol were included. A total of 79 were female (56.8%) and the majority had a B‐type phenotype 130 (93.5%). A complete response (CR) was achieved in 64.3%. Relapse was confirmed in 52.7% of those who obtained CR. Negative minimal residual disease (MRD) was achieved in 20% of patients. The median overall survival (OS) was 11 months (95% CI: 7.49–14.50) with a 5‐year OS of 14.0%. A total of 12 (8.6%) deceased during induction phase.

**Conclusion:**

Adult patients treated with the Hyper‐CVAD protocol in Ecuador achieve rates of CR, MRD, and OS lower than those presented in international cohorts, as well as higher rates of treatment‐related toxicity.

## 1. Introduction

The Hyper‐CVAD protocol is a chemotherapy widely used in hematological malignancies. This regimen of treatment consists of hyperfractionated cyclophosphamide, vincristine, doxorubicin, and dexamethasone in alternation with high dose of methotrexate and cytarabine [1]. The protocol was initially designed at MD Anderson Cancer Center, inspired by a pediatric protocol developed by St. Jude Children’s Research Hospital. At first, it faced great limitations in its initial design due to its safety, especially infectious complications, high induction mortality, and high rate of relapse [2]. Once modified and adapted to be used in adult patients, it has been established as a main option for the management of non‐Hodgkin lymphoma and leukemia [3–6].

Regarding leukemia management, the Hyper‐CVAD protocol, which does not contain L‐asparaginase, stands as one of the principal treatments of adults with acute lymphoblastic leukemia (ALL) [7]. ALL is a hematological neoplasia commonly found in pediatric patients with a low prevalence in the adult population. According to demographic studies, the prevalence of ALL decreases as the age increases. However, the mortality rate increases as the population ages [8, 9]. Therefore, the survival rate in adults ALL leukemia is considered significantly poor [10].

Currently, despite the ponderable advances in the ALL management, the mortality rate of ALL in adults is highly disproportional in comparison to pediatric protocols. Additionally, there are limited treatment options for adults; furthermore, almost all available regimens of treatment are pediatric‐designed protocols containing L‐asparaginase, except for Hyper‐CVAD. Noticeably, the Hyper‐CVAD protocol presented a good complete response (CR) rates in large clinical trials and 5‐year overall survival (OS) rate, comparable with L‐asparaginase‐based protocols [11, 12]. Outcomes in these studies can vary depending on medical and technological availability of the treatment center, patient demographic characteristics, and socioeconomic differences, as well as the exclusion criteria proposed. Therefore, the aim of this study is to determine the outcomes of adults with ALL treated with the Hyper‐CVAD protocol in the Ecuadorian population.

## 2. Population and Methods

### 2.1. Study Design

A multicenter, retrospective, cross‐sectional study was performed in patients older than 15 years old with a diagnosis of ALL registered between January 2015 and December 2022 in 8 specialized centers in Ecuador. All patients meeting the inclusion criteria were retained in the study cohort; no individuals were excluded from the study overall.

### 2.2. Diagnostic and Classification Criteria

Diagnosis of ALL was established according to the World Health Organization 2016 criteria [13]. Electronic medical records were reviewed for all patients to register complete blood count (CBC) prior to diagnosis and bone marrow aspiration needle as well as bone marrow biopsy. The type of ALL was defined based on immunohistochemistry or immunophenotyping techniques.

We registered the following data as well as the diagnostic techniques used: karyotype, BCR::ABL translocation performed by fluorescence in situ hybridization (FISH) or polymerase chain reaction (PCR), KTM2A rearrangement by FISH, and central nervous system (CNS) infiltration by conventional cytology or flow cytometry (FC). Risk stratification was conducted using MRC UKALL XII/ECOG E2993 trial parameters: age, CBC, and SNC infiltration. Patients were classified as high risk if age ≥ 35 years old, initial white blood count (≥ 30 × 10^9^ B linage and ≥ 100 × 10^9^ T linage), and/or SNC infiltration at diagnosis; those who lacked these criteria were classified as standard risk [12].

We divided patients by age groups based on the National Cancer Institute Adolescent and Young Adult (AYA) Oncology Progress Review Group: individuals between 15 and 39, 40 and 64, and ≥ 65 years old [14, 15].

### 2.3. Treatment Response Assessment

To establish a response to treatment, we used the CR and minimal residual disease (MRD) posterior to the induction phase, relapse, 5‐year OS, median OS, and median disease‐free survival (DFS). CR was defined based on Cheson criteria, which are characterized by the absence of extramedullary disease, the absence of peripheral blasts, < 5% of blasts in a bone marrow biopsy, white blood count (WBC) ≥ 1.5 × 10^9^/L, and platelet ≥ 100 × 10^9^/L [16]. The MRD was determined by FC, with a cutoff value of < 0.01, which is equal to 1 cell per every 10,000 cells among all mononuclear cells in a bone marrow sample [17, 18]. Relapse was registered in patients who achieved CR and posteriorly presented any of the European Working Group for Adult ALL (EWALL) consensus criteria: evidence of blasts 5% in a bone marrow sample and/or presence of blasts in a peripheral blood smear and/or extramedullary disease [19]. Median DFS was defined as the time from diagnosis until patient’s relapse, death, or last follow‐up, whichever comes first. Methotrexate serum levels were not measured during the treatment with the Hyper‐CVAD protocol.

Efficacy of Hyper‐CVAD was measured by 5‐year OS and median OS analysis. Both measures were calculated from the time of diagnosis until death or last medical appointment registered. Added to this, we examined treatment‐related mortality.

### 2.4. Statistical Analysis

Data were described as the mean or median for continuous variables based on its distribution, and categorical variables were presented as frequencies or percentages. Absolute numbers as well as the proportion of individuals within each group were presented in crosstabs, and the differences between groups were calculated using the Chi‐square test, *t*‐test, and Fisher exact test.

Descriptive analysis was performed across relevant variables, including age, sex, WBC at diagnosis, risk stratification, and CNS infiltration. Analyses of the factors impacting CR and MRD were not conducted because of the low number of tested individuals, disproportion between groups, and, in some instances, absence of subjects in specific subgroups. Subgroups with very limited representation, such as T‐phenotype ALL, KMT2A rearrangement, and transplanted subjects, were excluded from the survival analysis.

In the survival analysis, the Kaplan–Meier method was used to estimate median and 5‐year OS. To assess the effect of covariates, analyses were performed within a univariate framework by stratifying the population according to relevant descriptive variables (e.g., age, sex, and treatment protocols), and comparisons between groups were performed using the log‐rank test. Multivariate analysis was not feasible due to limited subgroup sample sizes and incomplete testing data (e.g., MRD, KMT2A rearrangement, and BCR::ABL1 status). Relapse was measured using cumulative incidence function with death as competitor, and the fine‐gray model was performed to estimate risk factors significantly related to relapse risk.

The statistical analysis was executed by SPSS 25 Version program, and the level of significance used in this study was 5%.

### 2.5. Ethical Approval

This study was approved by the Humans Being Investigation Ethical Committee of Manabí’s Technical University with the following approval code: CEISH‐UTM‐EXT_23‐01‐5_BESV. During the execution of this study, no personal information was recorded, and the confidentiality of the data was preserved. The study received financial support from Varifarma, Ecuador.

## 3. Results

### 3.1. Demographic Characteristics

A total of 139 patients with a diagnosis of ALL treated with the Hyper‐CVAD protocol were included. Most ALL subjects had B‐type phenotype 130 (93.5%) and 9 (6.5%) had T‐type phenotype. A total of 79 (56.8%) were women. Median age at diagnosis was 37 (IQ: 24–50) with 77 (55.4%) younger than 40 years old. A total of 129 subjects were eligible for risk stratification, 96 (74.4%) were characterized as high risk, and 33 (25.6%) as standard risk. Conventional cytogenetics was performed in 112 (80.6%) with normal results found in 46 (41.1%). Also, BCR::ABL translocation was evaluated in 95 (68.30%) with 25 (26.3%) cases with positive results. The KTM2 rearrangement was analyzed in 27 (19.4%) patients and 7 (25.9%) of these showed a positive result. The remaining of demographic characteristics of the patients is presented in Table 1.

**Table 1:** Baseline characteristics of the study population.

Variable	Value	*n*	(%)
Median age at diagnosis	37 (IQ: 24–50)		

Sex	Female	79	56.8
	Male	60	43.2

Cell lineage	B‐type ALL	130	93.5
	T‐type ALL	9	6.5

Age group	15–39	77	55.4
	40–64	54	38.8
	65 or older	8	5.8

WBC at diagnosis	< 30 × 10^9^/L	90	64.7
	≥ 30 × 10^9^/L–< 100 × 10^9^/L	26	18.7
	≥ 100 × 10^9^/L	23	16.6

Risk stratification^∗^	Standard risk	33	25.6
	High risk	96	74.4

BCR::ABL translocation^∗^	Negative	70	73.7
	Positive	25	26.3

KTM2 rearrangement^∗^	Negative	20	74.1
	Positive	7	25.9

Karyotype^∗^	Normal	46	44.1
	Other CGT abnormalities	31	27.7
	Too few metaphases	19	16.9
	High hyperdiploidy	11	9.82
	Complex	3	2.68
	Low hypodiploidy	2	1.78

CNS infiltration^∗^	Negative	86	77.5
	Positive	25	22.5

*Note:* IQ, interquartile.

Abbreviation: CNS, central nervous system.

^∗^Percentages are reported in relation to the number of subjects with available evaluations.

### 3.2. Treatment Evaluation

A total of 74 out of 115 patients (64.3%) achieved CR, and relapse was confirmed in 39 out of 74 (52.7%) of those who obtained CR. Also, negative MRD was achieved in 20 (20.00%) of the patients.

A total of 101 (72.7%) patients treated with the Hyper‐CVAD protocol died. In the descriptions of the causes of death, 12 (8.6%) and 15 (10.8%) died in induction and consolidation phases, respectively. A total of 71 (51.1%) of deaths were caused by progression of the disease, and 3 deaths were caused by events not related to treatment or disease.

### 3.3. T‐Phenotype ALL

A small number of patients with T‐type ALL were included in our study. A total of 8 out of the 9 patients (88.8%) died. Amongst the determined causes, 6 (75%) subjects died due to disease progression. A total of 4 out of 8 patients evaluated achieved CR, and all 4 suffered relapse. One out of 4 (25%) tested achieved negative MRD.

### 3.4. Hematopoietic Stem Cell Transplant (HSCT)

Only 3 patients underwent HSCT, of which 2 were alive at the end of the study.

### 3.5. Relapse

**Figure 1:**
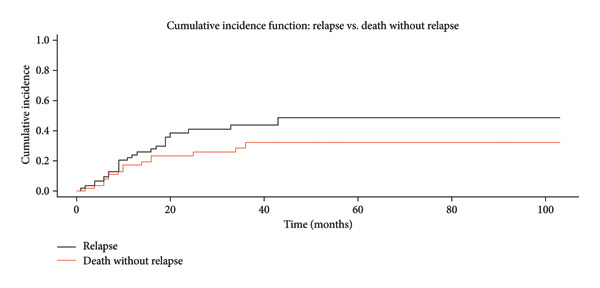
Cumulative incidence function: cumulative incidence curves for relapse and nonrelapse mortality (NRM).

Relapse was recorded in 39 (52.7%) patients. Median and 5‐year relapse‐free survival (RFS) was 16 months (95% CI: 12–25) and 20%, respectively. Cumulative incidence for relapse (CIR), considering death as a competitor event at 60 months, was 48.4% (95% CI: 32.4%–64.5%), while the nonrelapse mortality (NRM) was 31.5% (95% CI: 18.2%–44.9%) (Figure 1). The AYA group was associated to 40% (95% CI: 0.18–0.88, *p* = 0.023) lower relapse compared with 40 years old and older individual, even when accounting for death as a competing event.

### 3.6. Survival Analysis

**Figure 2:**
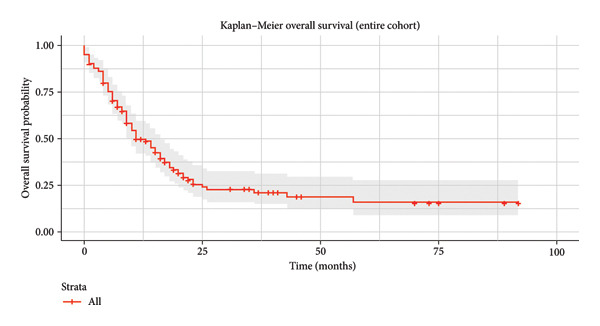
Kaplan–Meier: OS of ALL patients treated with the Hyper‐CVAD protocol.

Median OS was 11 months (95% CI: 7.49–14.50) and a 5‐year OS of 14% (Figure 2).

**Figure 3:**
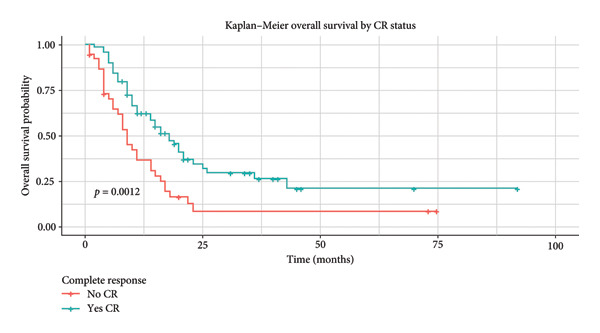
Kaplan–Meier: OS of patients by CR.

**Figure 4:**
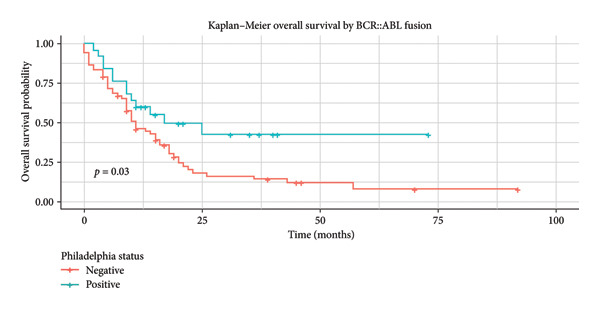
Kaplan–Meier: OS of patients by BCR::ABL translocation.

**Figure 5:**
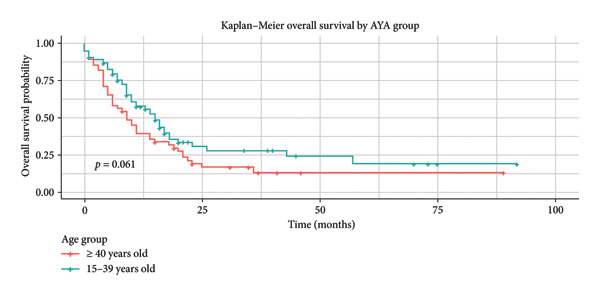
Kaplan–Meier: OS of patients by the AYA group.

Patients who achieved CR reached a significant median OS of 16 months (95% CI: 11.02–20.91), with a 5‐year OS of 19.3% (Figure 3). Phi + status was significantly associated to better median OS, 17 months (95% CI: 0.0–35.07), and a 5‐year OS of 42.4% (Figure 4). In the AYA group, mean OS was 15 months (95% CI: 9.45–18.54), with a 5‐year OS of 19.2% (Figure 5).

**Figure 6:**
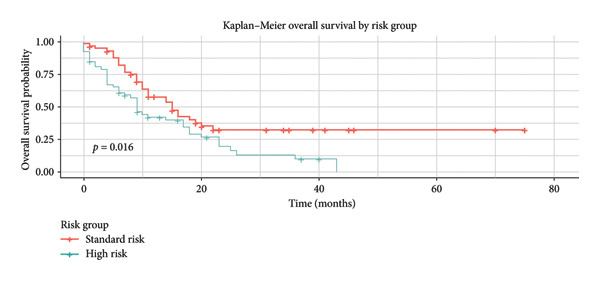
Kaplan–Meier: OS of patients by the risk group.

Regarding risk stratification, patients classified as high risk had a significant lower median OS of 9 months (95% CI: 7.05–10.94), with a 5‐year OS of 12.7% (Figure 6). A detailed analysis of prognostic factors influencing survival in patients treated with the Hyper‐CVAD protocol is presented in Table 2.

**Table 2:** Univariate analysis of ALL patients treated with the Hyper‐CVAD protocol.

Variable	*n* (%)	Median OS in months	5‐year OS	*p* value
AYA group				
15–39 years	77 (54.4)	15 [95% CI: 9.45–18.54]	19.2%	*p* = 0.061
≥ 40 years	54 (38.8)	9 [95% CI: 5.48–12.51]	13.4%	
Sex				
Female	79 (56.8)	13 [95% CI: 8.71–17.28]	12.2%	*p* = 0.723
Male	60 (43.2)	11 [95% CI: 7.61–12.39]	19.2%	
Risk group: High				
Standard	96 (74.40%)	9 [95% CI: 7.05–10.94]	12.7%	*p* = 0.016
CR	33 (25.60%)	16 [95% CI: 13.84–18.15]	32.8%	
No	41 (35.60)	9 [95% CI: 7.28–10.71]	8.3%	*p* = 0.0012
Yes	74 (64.40)	16 [95% CI: 11.08–20.91]	19.3%	
Negative MRD: No				
Yes	80 (80.00)	15 [95% CI: 9.62–20.37]	14.6%	*p* = 0.940
Relapse: No	20 (20.00)	10 [95% CI: 7.20–12.79]	21.4%	
Yes	35 (47.29)	12 [95% CI: 7.25–16.86]	55.6%	*p* ≤ 0.001
BCR::ABL fusion	39 (52.70)	11 [95% CI: 6.70–15.79]	3%	
Negative	70 (73.70)	11 [95% CI: 7.21–14.78]	7.4%	*p* = 0.030
Positive	25 (26.30)	17 [95% CI: 0.0–35.07]	42.4%	
CNS infiltration at debut				
No	86 (77.50)	11 [95% CI: 7.28–14.71]	21.7%	*p* = 0.231
Yes	25 (22.50)	9 [95% CI: 6.36–11.63]	12.5%	
# of leukocytes/mm^3^ at debut				
< 30.000	90 (64.70)	14 [95% CI: 10.19–17.80]	23.7%	*p* = 0.040
30.000–100.000	26 (18.70)	8 [95% CI:4.26–11.73]	0.00%	
> 100.000	23 (16.50)	9 [95% CI: 0.40–17.53]	0.00%	

*Note:* Analyzing factors impacting OS in ALL treated with the Hyper‐CVAD protocol, we observed a significant difference for the following factors: standard group (16 vs. 9 months; *p* = 0.016), complete response (16 vs. 9 months; *p* = 0.002), relapse (11 vs. 12 months; *p* ≤ 0.001), BCR::ABL translocation (17 vs. 11 months; *p* = 0.025), and WBC below 30,000/μL at diagnosis (14 vs. 8 vs. 9 months; *p* = 0.040).

Abbreviations: AYA, Adolescent and Young Adult; CNS, central nervous system; CR, complete response; MRD, minimal residual disease.

Median and 5‐year DFS for total cohort was 11 months (95% CI: 10–16) and 12.6%, respectively. DFS univariate analysis showed statistically significant differences: AYA group was 16 months (95% CI: 10–24) versus 9 months (95% CI: 6–12) non‐AYA group, *p* = 0.004. CR was 16 months (95% CI: 11.9–25) vs. 9 months (95% CI: 7–15) refractory patients, *p* = 0.007. Phi+ (BCR::ABL fusion) was 25 (95% CI: 10‐NA) vs. 10 months (95% CI: 9–15) Phi‐subjects, *p* = 0.010.

## 4. Discussion

Treatment of ALL in adults represent a significant challenge due to differences in demographic, clinical, and genetics factors. Factors like higher numbers of comorbidities and a lower bone medullary reserve influence the poorer outcomes seen in adults ALL vs. pediatric population [20, 21]. These differences are evidenced among the various available pediatric protocols but also are observed when particular protocols is applied in two different demographic populations.

First, taking in consideration the results observed at MD Anderson Cancer Center, in which 204 subjects with a median age of 39.5 years were studied between 1992 and 1998, a CR of 91%, a 5‐year OS of 38%, and long‐term follow‐up with similar results were determined [4, 11]. In our study, we evaluated 139 individuals with significantly lower CR and 5‐year OS rates (64.3% and 14%, respectively). Nevertheless, values in our cohort are even lower than those seen in other studies, as shown in retrospective studies of different countries with a CR between 81.7% and 84.2% [7]; a 5‐year OS of 50.1%–51.8% in Iran [22, 23]; CR of 84.2% and a 5‐year OS of 26.30% in Turkey [24]; CR of 83% was observed for Chinese [25]; CR of 73.70% for Mexico [26]; in a Brazilian report, a 93.8% CR and 5‐year OS of 35% was observed [27]; Jordan achieved a CR of 88% [28]; and in Saudi Arabia, a 5‐year OS 65.3% was observed [29]. It is also important to highlight that most of these studies evaluated populations smaller than 100 subjects and that OS was commonly reported at 5 years, although some studies used shorter follow‐up periods of 1 or 2 years (Table 3).

**Table 3:** Comparison of the outcomes with Hyper‐CVAD treatment in adults with acute lymphoblastic leukemia among the studies.

Year	Author	Country	Study design	Patients, n	CR % (*n*)	Induction‐therapy mortality % (*n*)	Relapse % (*n*)	OS % (years)	DFS (months)
2000	Kantarjian et al.	USA	Single center, retrospective	204	91% (185)	6% (15)	45% (85)	39% (5 years)	Unavailable
2004	Kantarjian et al.	USA	Single center, retrospective	288	92% (264)	5% (14)	54% (145)	38% (5 years)	Unavailable
2013	Buyukasik et al.	Turkey	Multicenter, retrospective	57	84.2% (48)	5.2% (3)	10.5% (6)	26.3% (5 years)	13.9 months
2014	Abbasi et al.	Jordan	Single center, retrospective	66	90% (59)	Unavailable	Unavailable	Unavailable	28 months
2014	Portugal et al.	Brazil	Single center, retrospective	49	93.8% (46)	6.1% (3)	50% (23)	35% (5 years)	21 months
2019	Yingying et al.	China	Single center, retrospective	30	83% (25)	Unavailable	Unavailable	Unavailable	Unavailable
2020	Almanza‐Huante et al.	Mexico	Multicenter, retrospective	137	73.7% (100)	8% (10)	60% (82)	28% (5 years)	Unavailable
2023	Salama et al.	Saudi Arabia	Multicenter, retrospective	45	65.3% (28)	Unavailable	Unavailable	Unavailable	Unavailable
2023	Malakoutikhah et al.	Irán	Single center, retrospective	70	84.28% (59)	Unavailable	50% (35)	61% (1 year) 42% (2 years)	13.3 months
2024	Present study	Ecuador	Multicenter, retrospective	139	64.4% (74)	8.6% (12)	52.7% (39)	14% (5 years)	11 months

Abbreviations: CR, complete response; DFS, disease‐free survival; OS, overall Survival; USA, United States of America.

Negative MRD had a median OS of 10 months and a 5‐year OS of 21.4%, whereas MRD‐positive cases demonstrated a median OS of 15 months with a 5‐year OS of 14.6%. This paradox likely reflects the small sample size of the MRD‐negative subgroup, where a few long‐term survivors disproportionately influenced the 5‐year estimate, and the larger MRD‐positive cohort, where early survival was longer but long‐term outcomes were less favorable. Negative MRD after the induction phase was observed in 20% of the population study. This finding is low compared with those found in international studies. In a study with negative Philadelphia chromosome treated with Hyper‐CVAD, negative MRD was found to be 58% [25]; another study that assessed treatment with Hyper‐CVAD on 18–49 years old subjects obtained a negative MRD of 84% [30]. In a study in Iraq, a negative MRD was reported in 62.1% [31].

Of those who achieved CR in our study, relapse was confirmed in 39 (52.70%). Even though this is considered high relapse rate, reports from some international cohorts describe similar rates ranging from 30%–35% (26), 40%–42% (4.24), to 50%–52% (4, 7, 11, 27). Treatment‐related toxicity, on the other hand, was found in 8.60% and 10.80% of the patients during the induction and consolidation phase, respectively. These values are worrisome indicators due to the fact that reports of mortality during the induction phase with Hyper‐CVAD are typically described to be less than 7%, even in similar low‐income countries, please see Table 3 [4, 11, 23, 24, 27, 28].

Baseline leukocyte count was found to be an important prognostic factor. Patients with a WBC below 30,000/μL at diagnosis demonstrated improved OS, with a proportion surviving beyond five years, whereas none of the individuals with higher WBCs survived to five years. This variable is included as a key determinant in the risk stratification model.

This study has limitations due to its retrospective and cross‐sectional nature. There were lack of data in some patients present in this study. We do not register Philadelphia chromosome‐positive patients who used tyrosine kinase inhibitors. However, Philadelphia chromosome‐positive patients presented better results compared with negative ones. It is also remarkable the lack of information regarding methotrexate serum levels during treatment, which could have affected the likelihood of obtaining CR and negative MRD. Risk stratification was not performed in 10 patients due to the absence of CNS infiltration evaluation at the time of diagnosis. Despite these limitations, the present study conveys important details regarding treating in a low‐income country environment, among which are the poor efficacy of the treatment, worse OS, and higher treatment‐related mortality when compared with international studies.

Our cohort may be explained by multiple factors. Possible contributors include delays in diagnosis and initiation of therapy, limited supportive care resources (e.g., restricted access to prophylaxis, blood products, and intensive care), delay in treatment delivery across centers, and socioeconomic barriers that may lead to treatment interruptions or reduced adherence.

It is important to consider the development of longitudinal, prospective studies to elucidate the causes or factors that cause worse outcomes in Ecuadorian adults with ALL treated with the Hyper‐CVAD protocol so that the results can be compared with countries with similar socioeconomic conditions.

## 5. Conclusion

Adult patients with ALL treated with the promising Hyper‐CVAD protocol in Ecuador presented low CR, OS, and MRD rates compared with international cohorts, as well as greater treatment toxicity–related events.

## Ethics Statement

This study was approved and authorized by the Human Research Ethics Committee of the Technical University of Manabí.

## Consent

Our study was classified as posing minimal risk in humans, and informed consent to access patient’s information was authorized by each of the participating hospitals.

## Conflicts of Interest

The authors declare no conflicts of interest.

## Funding

This study received funding from the Varifarma pharmaceutical company for design and execution.

## Data Availability

The datasets used for the current study are available from the corresponding author upon reasonable request.
